# Next generation editors

**DOI:** 10.1093/jxb/erad037

**Published:** 2023-03-13

**Authors:** John E Lunn

**Affiliations:** Max Planck Institute of Molecular Plant Physiology, D-14476 Potsdam-Golm, Germany

**Fig. 1.**
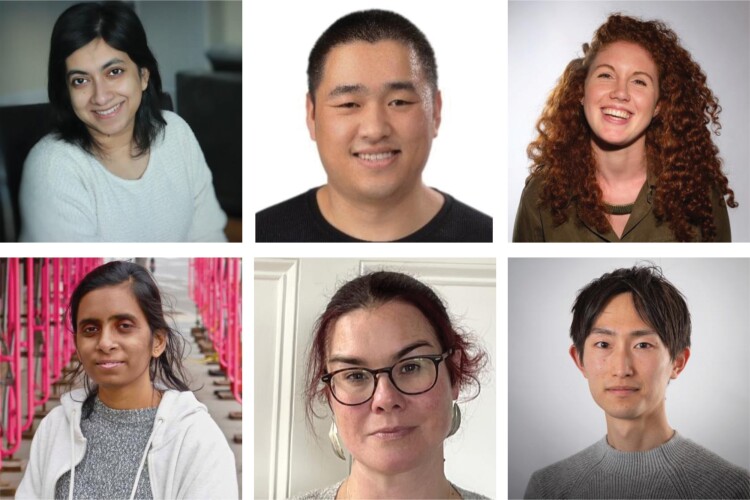
Introducing *JXB’*s Editorial Interns. Top (L to R): Nishat Shayala Islam, Chao Su, Francesca Bellinazzo. Bottom (L to R): Arpita Yadav, Catherine Walsh, Konan Ishida.

**The *Journal of Experimental Botany* is pleased to announce the appointment of six early career researchers as editorial interns: Francesca Bellinazzo (Wageningen University and Research, the Netherlands), Konan Ishida (University of Cambridge, UK), Nishat Shayala Islam (Western University, Ontario, Canada), Chao Su (University of Freiburg, Germany), Catherine Walsh (Lancaster University, UK), and Arpita Yadav (University of Massachusetts Amherst, MA, USA) (Fig. 1). The aim of this programme is to help train the next generation of editors.**

Each intern has been paired with one of our Associate Editors as a mentor, who will guide them through all aspects of the editorial process for selected manuscripts: initial editorial assessment, selection of reviewers, and evaluation of the reviews to reach a decision. The strict rules of editorial confidentiality will apply at all times, and the final decision on each manuscript will remain the sole responsibility of the Associate Editor. Our interns will also be invited to write Insight commentaries on selected papers and contribute to the journal’s social media output. If you would like to learn more about our editorial interns, please visit the *JXB* website.

As we begin a new year, we look forward with hope to an end to the Covid-19 pandemic, which has had a major impact on all of our lives, both personal and professional, and *JXB* has been no exception. Our editorial staff in the Lancaster office and members of our editorial board rose magnificently to the challenges during these difficult times, guided by our goal to handle all submissions in an efficient, constructive, and courteous way, and helping to maintain the high publishing standards the plant science community expects from us. We are pleased to report a further increase in our Clarivate™ Web of Science™ impact factor to 7.378, reflecting the high quality of the manuscripts we receive. We would like to thank all the authors who entrusted their work to us for publication and our valued reviewers who kindly gave their time and the benefit of their expert knowledge to support *JXB*.

*JXB* was founded by the Society for Experimental Biology (SEB) and remains part of the SEB family of journals that support its mission to promote science, supporting the science community through its scientific meetings, travel grants, careers workshops, and public outreach and education programmes. This year, the SEB celebrates the centenary of its founding in 1923 (https://www.sebiology.org/centenary.html), and we congratulate the society, its officers and members, past and present, for this remarkable achievement. We are looking forward to an exciting programme of plant, animal, and cell science at the SEB Centenary Conference in Edinburgh (4–7 July 2023; https://www.sebiology.org/centenary/centenary-conference.html), where there will be an opportunity to meet some of our editors and editorial staff. To mark the occasion, we have commissioned a special series of SEB Centenary Reviews, to be published later this year, representing the diverse aspects of plant science published by *JXB*. We thank the eminent scientists who kindly agreed to contribute to this collection, and we hope that you will enjoy reading the reviews and that you will continue helping *JXB* to support the invaluable work of the SEB in its second century.

